# Production, Signaling, and Scavenging Mechanisms of Reactive Oxygen Species in Fruit–Pathogen Interactions

**DOI:** 10.3390/ijms20122994

**Published:** 2019-06-19

**Authors:** Ying Wang, Dongchao Ji, Tong Chen, Boqiang Li, Zhanquan Zhang, Guozheng Qin, Shiping Tian

**Affiliations:** 1 Key Laboratory of Plant Resources, Institute of Botany, Innovation Academy for Seed design, Chinese Academy of Sciences, Beijing 100093, China; hswangying@sina.cn (Y.W.); jidongchao@ibcas.ac.cn (D.J.); chentong@ibcas.ac.cn (T.C.); bqli@ibcas.ac.cn (B.L.); zhangzhanquan82@ibcas.ac.cn (Z.Z.); gzqin@ibcas.ac.cn (G.Q.); 2 University of Chinese Academy of Sciences, Beijing 100049, China; 3 Key Laboratory of Post-Harvest Handling of Fruits, Ministry of Agriculture, Beijing 100093, China

**Keywords:** reactive oxygen species, fruit, defense response, fungal pathogen, virulence

## Abstract

Reactive oxygen species (ROS) play a dual role in fruit–pathogen interaction, which largely depends on their different levels in cells. Fruit recognition of a pathogen immediately triggers an oxidative burst that is considered an integral part of the fruit defense response. ROS are also necessary for the virulence of pathogenic fungi. However, the accumulation of ROS in cells causes molecular damage and finally leads to cell death. In this review, on the basis of data regarding ROS production and the scavenging systems determining ROS homeostasis, we focus on the role of ROS in fruit defense reactions against pathogens and in fungi pathogenicity during fruit–pathogen interaction.

## 1. Introduction

Postharvest diseases induced by fungal pathogens are the principal causes for fruit decay, which leads to tremendous economic losses annually [1]. In the case of pathogen infection, fleshy fruits rely on their own innate immune capacity to resist pathogen attack [2]. Excessive reactive oxygen species (ROS) production in response to unfavorable conditions, also known as oxidative burst, has been recognized as one of the earliest induced defense responses in plants [3]. This production of ROS is biphasic: the first phase usually occurs within minutes after pathogen attack but is transient and weak, whereas the second phase is much more intense and sustained, lasting for several hours [4]. However, overproduction of ROS causes impairments in DNA, lipids, and protein, eventually leading to cell death and progressive aging of an organism [5,6,7]. Generally, senescent fruits always display higher susceptibility to pathogen attack, and, in turn, senescence and decay are accelerated in infected fruit [8]. For pathogens, ROS also play an important role in their infection processes, and the lack of ROS-producing systems can affect fungal toxicity and their interaction with plants [9,10,11]. During this interaction, pathogens may encounter ROS generated by the host and, as a result, they may be directly killed. On the other hand, cell death caused by ROS may lead to cellular necrosis in the hosts, from which quiescent pathogens (hemibiotrophic or necrotrophic) acquire nutrients, switching into the devastating necrotrophic life mode [1,12]. In order to cope with oxidative stress, both plants and pathogens have evolved efficient scavenging systems to modulate ROS homeostasis, which eventually determine the incidence, development, and consequences of diseases in plants [3,13]. Considerable progress has been made in understanding the mechanisms regulating plant–pathogen interactions. Here, we mainly focus on the current advances in the study of fruit–pathogen interactions mediated by ROS, which may broaden our understanding of the role of ROS in fruit defense and fungal pathogenicity.

## 2. ROS Production Sites and Scavenging Systems

ROS, such as superoxide anion (O_2_^·-^), hydroxyl radical (·OH), and hydrogen peroxide (H_2_O_2_), are byproducts of normal metabolism in cells [4]. There are several enzymatic systems involved in apoplastic ROS production following successful recognition of phytopathogenic fungi, including glucose peroxidase, xanthine oxidase, and different classical plant peroxidases [3]. Among them, membrane-resident NADPH oxidase is one of the major factors generating ROS during plant–pathogen interaction [11,14,15]. NADPH oxidases are transmembrane proteins catalyzing superoxide production by transferring electrons from intracellular NADPH to molecular oxygen in the apoplast [16,17]. Superoxide is further converted to H_2_O_2_ either by spontaneous dismutation or by the catalytic activity of a cell wall superoxide dismutase [18]. Noteworthy, since non-invasive imaging directly applicable for detecting ROS in fruits is still unavailable, no result has been reported for ROS detection in living fruit tissues until now. Therefore, the current knowledge of ROS production and scavenging in plants has been mainly obtained from non-fruit tissues; however, it can nonetheless offer insights about ROS production in fruits.

**Figure 1:**
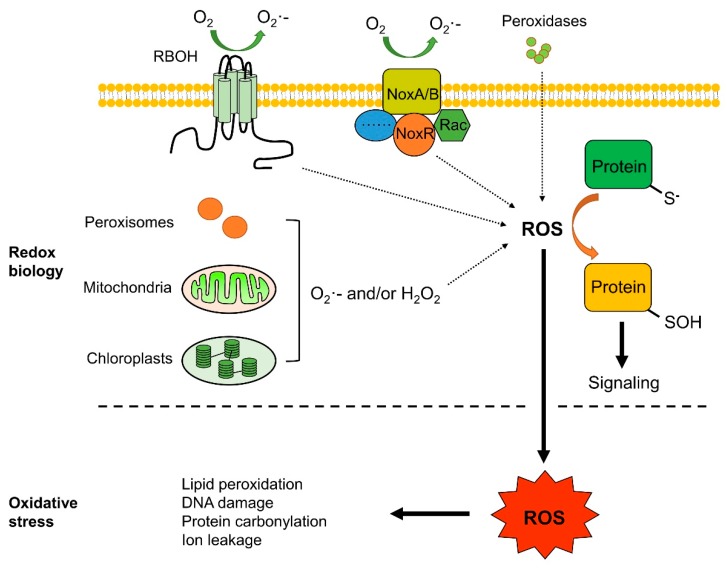
Generation sites of reactive oxygen species (ROS) and redox biology. ROS are produced by respiratory burst oxidase homologs (RBOHs), mitochondria, chloroplasts, peroxisomes, and cell wall-resident peroxidases (PER). Subsequent H_2_O_2_ accumulation may oxidize cysteine residues in proteins, affect their redox states and functions, and regulate related signaling pathways. Excessive ROS may lead to oxidative stress, which may cause lipid oxidation, DNA damage, protein carbonylation, and injuries to other cellular components.

Although the oxidative burst in fruit after pathogen recognition mainly occurs in the apoplast, ROS produced in other cellular compartments may also contribute to defense signals [2]. Mitochondria, chloroplasts, and peroxisomes are the main potential sources of ROS during biotic responses (Figure 1) [19,20]. ROS produced in the mitochondria are tightly associated with the electron transport chain (mETC), which is located in the inner mitochondrial membrane. Thylakoid, harbored in chloroplast, is the main site of chloroplastic ROS generation, which is closely associated with light-dependent photosynthetic reactions [21]. Besides chloroplasts and mitochondria, peroxisomes are also major sources of intracellular ROS [20], serving as monolayer-membrane organelles with multiple metabolic functions. 

Oxidative stress is caused by unfavorable ROS levels in the environment or even by normal metabolic processes (Figure 1). Therefore, many organisms have evolved ROS scavenging systems, that can be enzymatic or non-enzymatic, enabling cells to maintain a non-toxic and steady-state level of ROS. The enzymatic ROS scavenging system is composed of superoxide dismutases (SODs), peroxidases (PODs), catalases (CATs), ascorbate peroxidase (APX), and glutathione peroxidase (GPX). SODs act as soon as ROS are generated and dismutate superoxide to H_2_O_2_, whereas CAT, APX, and GPX subsequently convert H_2_O_2_ to H_2_O [3,21]. Non-enzymatic antioxidants, such as ascorbate, glutathione (GSH), flavonoids, tocopherol, and alkaloids, are also major cellular redox buffers. GSH is a ubiquitously distributed thiol-containing antioxidant in cells, which may be converted to glutathione disulfide (GSSG, an oxidized form) by ROS, using NADPH as the electron donor [21]. ROS scavenging systems are essential for managing ROS levels both in plants and in pathogens. It is worth emphasizing that the destructive, protective, or signaling role of ROS in cells depends on the complex equilibrium between ROS production and scavenging at appropriate time and sites.

## 3. Roles of ROS in Regulating Fruit Defense Responses

### 3.1. Antioxidants Participate in Fruit Defense Responses

During the interactions between plants and pathogens, sequential cellular, biochemical, and molecular changes occur in plant responses against pathogens [22]. Some studies have shown that antioxidants play key roles in inhibiting fruit senescence [23,24]. Conversely, the oxidative damage in mitochondrial proteins caused by ROS accumulation can accelerate fruit senescence [25,26]. Comprehensive studies on antioxidant enzymes in the citrus fruit infected by *Penicillium digitatum* showed that the antioxidant activities of CAT, SOD, and APX decreased during orange–*P. digitatum* interaction. In non-infected areas of the flavedo, all enzymes displayed higher activities, which may be related to the high resistance of the flavedo to pathogen infection [27]. Similar to the results mentioned above, a transcriptomic analysis of apple fruit in response to *Penicillium expansum* infection indicated that genes encoding ROS-detoxifying enzymes, such as SOD, APX, and POD, were significantly upregulated [28]. In an attempt to probe the antimicrobial mechanisms, exogenous substances, such as oxalic acid [29], trisodium phosphate [30], rhamnolipids [31], methyl thujate [32], chitosan [33], and biocontrol yeasts [34], were employed to enhance fruit resistance to postharvest fungal pathogens, which resulted in significantly decreased disease severity. These substances also increased the activity of antioxidant enzymes (POD, SOD, CAT), activated the expression of related genes, improved the ROS-scavenging capacity, and further decreased ROS levels in the treated fruit samples. Current evidence indicates that silencing *SlPL*, the gene encoding a pectate lyase in tomato, results in increased activities of CAT, SOD, and POD in *SlPL*-RNAi-treated fruit and reduces the susceptibility of tomato fruit against *Botrytis cinerea* [35]. In general, these results further confirm the importance of antioxidant enzymes in balancing cellular ROS and enhancing the ability of fruit to withstand fungal pathogens.

### 3.2. ROS–Phytohormone Crosstalk

**Figure 2:**
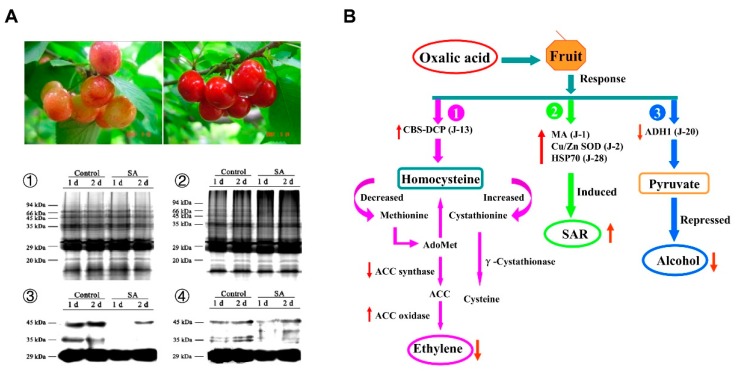
ROS is involved in the responses to salicylic acid (SA) and oxalic acid by modulating protein carbonylation, ethylene biosynthesis, and alcohol dehydrogenase (ADH) by-pass [29,41]. After inoculation with *Penicillum expansum*, less carbonylated proteins were accumulated in SA-treated sweet cherry fruit than in control fruit (**A**), whereas ROS acted synergistically with ethylene biosynthesis/signaling and ADH by-pass in the responses to oxalic acid (**B**). CBS-DCP: CBS domain-containing protein (J-13); MA: major allergen (J-1); Cu/Zn SOD: Cu/Zn superoxide dismutase (J-2); HSP70: heat shock protein 70 (J-28); ADH1: alcohol dehydrogenase 1 (J-20); PDH E2: dihydrolipoamide acetyltransferase (E2) of pyruvate dehydrogenase complex (J-14); AdoMet: S-adenosylmethionine; ACC: 1-aminocyclopropane-1-carboxylic; SAR: systemic acquired resistance.

A subtle interplay between ROS and phytohormones, such as salicylic acid (SA), jasmonic acid (JA), and ethylene (ET), has been documented in the interactions between fruit and pathogens [36,37]. In a recent transcriptomic analysis identifying genes whose expression correlated either positively or negatively with L-ascorbic acid content in tomato fruits, cluster analysis using Self-Organizing Tree Algorithm (SOTA) showed that the genes related to hormone signaling, which are dependent on the oxidative status of the fruit, were modulated in relation to L-ascorbic acid content in tomato [36] (Figure 2). Moreover, it has been revealed that SA could protect fruits against pathogenic fungi [38,39]. SA improved the resistance of sweet cherry fruit to *P. expansum* [40,41] and of pear fruit to *Alternaria alternata* [42] by inducing the activity of anti-oxidant enzymes and pathogenesis-related proteins. Moreover, SA application alleviated disease severity in postharvest citrus fruit by inducing the accumulation of H_2_O_2_, primary metabolites, and lipophilic polymethoxylated flavones [43]. However, SA may also facilitate H_2_O_2_ accumulation during the oxidative burst induced by infection with virulent pathogens [44]. A recent study pointed out that acibenzolar-S-methyl (ASM) treatment could enhance the activity of NADPH oxidase and accelerate the production of H_2_O_2_ in muskmelon, indicating the importance of ROS in ASM-induced resistance in muskmelon [45].

JA plays a prominent role in plant defense response through prompt metabolization to methyl jasmonate (MeJA) [46,47]. Tomato fruit treated with exogenous MeJA display a significantly decreased diameter of gray mold lesion caused by *B. cinerea*, which may be attributed to H_2_O_2_ accumulation, elicitation of antioxidative reaction, and prevention of protein carbonylation in fruit [48]. MeJA treatment also increases the activities of chitinase, β-1,3-glucanase, and POD in peach fruit, and further induces high resistance against *Monilinia fructicola* and *P. expansum* [49]. Usually, MeJA-treated fruits show an H_2_O_2_ burst and the accumulation of phenolic compounds, such as lignin and phytoalexin, which is beneficial for fruit defense responses.

The roles of ET in defense responses of plants are diversified and depend on the crosstalk with ROS [8,50]. As an inhibitor of ET perception, 1-methylcyclopropene (1-MCP) has been widely used to maintain fruit quality during postharvest storage via a decrease of ethylene production and induces the activities of enzymes involved in ROS scavenging such as PPO, CAT, and SOD [50,51,52]. Kiwifruits treated with conditioning combined with 1-MCP increased the fruit’s total antioxidant capacity and reduced the incidence rate of disease caused by *B. cinerea* [53]. Tomato fruits treated with tran-2-hexenal showed enhanced activities of antioxidant enzymes and elevated expression levels of genes encoding the ethylene receptor, which further alleviated the incidence of gray mold [54]. These results suggest that the controlling effect of trans-2-hexenal on gray mold may be related to ET/ROS-mediated systemic resistance. In addition, brassinosteroid treatment (BR) of tomato and cucumber at low concentration led to enhanced resistance against *Sphaerotheca fuliginea* and *B. cinerea* [55]. Furthermore, we found that BRs may alleviate jujube fruit decay by reducing ethylene production and scavenging ROS accumulation. The activities of several defense-related enzymes and antioxidant enzymes including phenylalanine ammonia lyase (PAL), CAT, and SOD in jujube fruit were significantly enhanced [56], which indicate a crosstalk between BRs, ET, and ROS during fruit–pathogen interactions. However, as most of the current understanding of ROS–phytohormone interactions is derived from non-fruit tissues, further confirmation is still required to draw parallels with fruits.

### 3.3. ROS–NO Reactions

Recent evidence suggests that nitric oxide (NO), a gaseous free radical, is an important intracellular signaling molecule involved in various physiological processes including growth and development, respiratory metabolism, maturation and senescence, as well as in responses to various stresses [57,58]. Following NO treatment, tomato fruits showed delayed ripening and increased activity of antioxidant enzymes in the late storage period, resulting in an increased resistance against *B. cinerea* [59]. An integrated signaling network involving NO and ROS was found in BcPG1-elicited grapevine defenses [60]. Exogenous NO treatment induced the accumulation of endogenous NO, H_2_O_2_, and O_2_^·-^ and increased the activity of NADPH oxidase, which contributed to increased resistance of peach fruit against *M. fructicola* [61]. However, H_2_O_2_ production was downregulated by NO, indicating that a feedback regulatory mechanism may exist between ROS and NO [62]. It was demonstrated that NO application could suppress spore germination of *P. expansum* and thus reduce its virulence on apple fruit [63], leading to the hypothesis that ROS may mediate the defense reactions of fruit by cooperation with NO. Interestingly, almost all major classes of plant hormones (SA, JA, ET, abscisic acid (ABA), and BRs) may influence, at least to some degree, the endogenous levels of NO [64]. The tomato mutant *sitiens* fails to accumulate ABA but exhibits an increase in NO and ROS production and has increased resistance to *B. cinerea* [65], suggesting a close relationship between NO and ABA, as well as the existence of ROS during fruit–pathogen interaction. These data suggest that a complicated network between ROS, NO, and phytohormones may function during fruit–pathogen interaction.

## 4. Roles of ROS in Fungal Development and Pathogenicity

### 4.1. Roles of NADPH Oxidases in Pathogens

**Figure 3:**
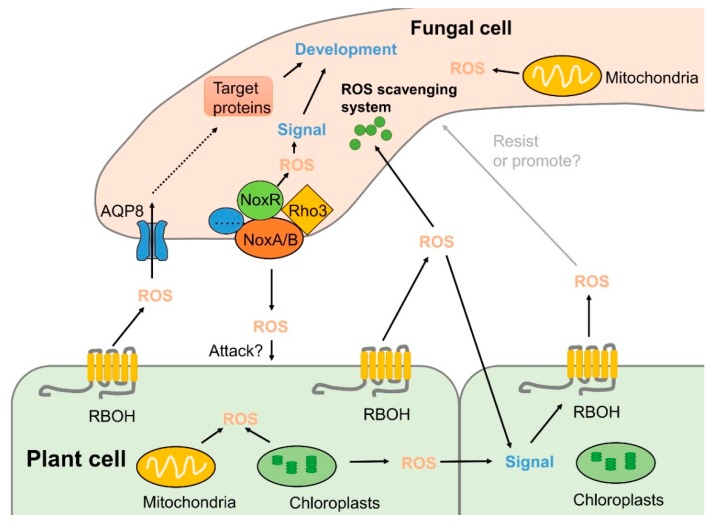
Inter-kingdom ROS signaling in the interaction between fungal pathogens and their host plants. Plant cells generate ROS by RBOHs in the plasma membrane and from several intracellular organelles upon pathogen recognition. In the meantime, fungal hyphae produce ROS by Nox complexes, mainly localized at the plasma membrane or endoplasmic reticulums (ER), which stimulate an oxidative burst response within the pathogen. Scavenging systems composed of enzymatic and non-enzymatic systems synergistically function to maintain intra- and extracellular redox homoeostasis in both plants and pathogens. Contents indicated by solid arrows are based on currently available experimental data, whereas those indicated by dashed arrow are based on hypotheses in literatures.

It has been clarified that ROS derived from NADPH oxidase (Nox) complex is involved in sexual differentiation and pathogenicity in many fungal species (Figure 3). Nox is a multi-subunit complex, and most fungi possess Nox homologs, i.e., NoxA (Nox1), NoxB (Nox2), and NoxC [66]. NoxA and NoxB are homologs of mammalian gp91^phox^ and are the best-characterized subunits that play key roles in various processes of fungal life, whereas fungal NoxC is closely related to the mammalian Nox5 and the plant RBOH enzymes, and its functions in fungi are still unclear [66,67]. In *B. cinerea*, both NoxA and NoxB are required for the development of sclerotia and full virulence. However, NoxB is needed for host penetration, whereas NoxA is related to post-infection hyphal growth [68]. Similar results have also been reported for other pathogens, such as *A. alternata* [66] and *Sclerotinia sclerotiorum* [69]. NoxR, encoding a homolog of the mammalian regulatory subunit p67^phox^, was shown to regulate both NoxA and NoxB in *B. cinerea* [68]. *ΔbcNoxR* and *ΔbcNoxAB* double-deletion mutants had the same phenotypes. *ΔbcNoxR* deletion mutant showed reduced growth rate, sporulation, and impaired virulence in apple, strawberry, and tomato fruits [11]. In *Aspergillus nidulans*, *NoxR* deletion mutant showed a similar phenotype to the *NoxA* mutant, resulting in loss of the ability to produce cleistothecia [70]. Moreover, the impairment in any of the *NoxA*, *NoxB*, or *NoxR* genes decreased the necrotic lesions on citrus cultivars compared to wild-type fruits [71]. NoxD, a homolog of the adaptor protein p22^phox^, is required for full function of the Nox complex and is found in *B. cinerea*, *Magnaporthe oryzae*, and *Podospora anserine* [67,72,73,74]. In addition, BcNoxD plays a key role in oxidative stress response [67]. Our study also showed that methyl thujate, an essential oil component derived from western red cedar, could stimulate ROS accumulation in the cytoplasm of *B. cinerea* hyphae and effectively control gray mold in apple fruit by upregulating the expression of genes encoding subunits of the Nox complex, such as *BcNoxB*, *BcNoxD*, and *BcNoxR* [32].

The small GTPase Rac and the proteins related to polarity establishment, BemA and Cdc24, are also important components of the fungal Nox complex [75]. Rac belongs to the Rho superfamily, which is activated by the GDP/GTP exchange factor (GEF) and binds to NoxR [1,76]. Increasing evidence has revealed that Rac has crucial functions during hyphal growth and development, and homologs of Rac have been identified in several filamentous fungi [77,78,79]. It was reported that a monomeric GTPase of the Rho superfamily (Rho3) in *B. cinerea* was involved in various cellular processes [10]. A *Δrho3* deletion mutant showed significant suppression of vegetative growth and conidiation compared to the wild-type (WT) strain. In addition, compared with the control, lesion development in tomato leaves and fruits and in apple was prominently repressed upon inoculation with conidia from the *Δrho3* mutant. Moreover, the *Δrho3* deletion mutant led to less ROS accumulation in hyphal tips of *B. cinerea* compared to the WT strain [10].

### 4.2. Effects of Antioxidants on Fungal Pathogenicity

The intracellular ROS level is crucial for developmental differentiation and virulence of many pathogenic fungi [80,81,82]. Fungal pathogens have developed robust antioxidation systems, including SODs, CAT, POD, glutathione, and thioredoxin, to eliminate ROS, are produced by the hosts during infection or as byproducts of the pathogens’ own aerobic respiration (Figure 3) [83,84]. In *Aspergillus niger*, *sodC* deletion led to excessive production of superoxide anion and increased content of H_2_O_2_. Moreover, a *ΔsodC* mutant had reduced virulence in Chinese white pear, indicating that *sodC* was crucial for the full virulence of *A. niger* during fruit infection [85]. Fungal CATs are also important antioxidant enzymes which catalyze the conversion of H_2_O_2_ to water and oxygen and are involved in fungal pathogenicity in plants [86,87]. Deletion of *cpeB*, a catalase-peroxidase encoding gene, resulted in a lower spore germination rate and slower lesion development in apple fruit, which contributed to increased sensitivity to H_2_O_2_ stress and suggested an essential role of *cpeB* for full virulence of *A. niger* during interactions with apples [88]. 

### 4.3. ROS Transport Affects Fungal Pathogenicity

Much progress has been made in the study of the production and scavenging systems of ROS in recent years, but it is still enigmatic how ROS are transported from their site of origin to their place of action or detoxification. As signaling molecules, the transport of ROS is closely related to their function [89]. Aquaporins (AQPs) are integral membrane proteins from the large water channel family functioning in water and/or glycerol transport. It has been previously documented that H_2_O_2_ transport is mediated by AQP isoforms in plants and mammals [90,91,92,93,94]. AQPs of plants are subdivided into seven groups, some of which have been proven to play an important role in plant disease processes [95,96,97]. In fungi, AQPs are classified into five groups, including two groups of classical AQPs and three groups of aquaglyceroporins [98]. It was demonstrated that, among the eight *AQPs*, only *AQP8* was involved in ROS production, distribution, and transport across membranes in *B. cinerea* [99]. An *AQP8* deletion completely inhibited the formation of conidia and infection structures in *B. cinerea* and impaired its ability to cause disease in tomato leaves and fruits. Interestingly, the expression of *NoxR* was significantly reduced in a *ΔAQP8* deletion mutant, suggesting that AQP8-based H_2_O_2_ transport may control the function of the Nox complex through influencing the expression of *NoxR* gene. Moreover, both *AQP8* and *NoxR* affect ROS distribution in the hyphal tips of *B. cinerea* [99], indicating the important role of *AQP8* in ROS transport and pathogenicity. 

## 5. Conclusions

In the light of recent advances, the importance of ROS in both hosts and pathogens during fruit–pathogen interactions has been fully addressed, and considerable progress has been made in the understanding of the complex metabolic machinery of ROS. In the present study, we reviewed the currently available information on the roles of ROS in the interaction between fruits and postharvest pathogens. Deducing from the fundamental results reported in non-fruit tissues, the oxidative burst, which occurs at the initial stage of the interaction, serves as one of the first defense lines in plants. The specific ROS levels in fruit or pathogen define their roles as signaling or harmful molecules. In the host plant, ROS act as a direct antimicrobial agent and contribute to host defense, whereas for pathogens, controlled production of ROS is essential for their development and full virulence. ROS also play a role in different signaling pathways as local or systemic diffusible second messengers. These results imply that the existence of ROS scavenging systems is necessary to maintain ROS homeostasis, which determines if ROS will act against pathogen or promote successful infection. However, it should be emphasized that fruits are highly specialized and unique to flowering plants, and their defense systems could behave quite differently from those of non-fruit tissues. Moreover, the developmental origins of fruit tissues in different fruiting plant families are also distinct, which may cause further differences in fruit tissues of distinct families. Therefore, the comparison of ROS signaling in fruit and other tissues may help answer several questions: are ROS signaling pathways are more specialized in fruit compared to non-fruit tissues? Do they involve different mechanisms or different sets of genes? What are the specific sensors of ROS and the immediate downstream pathways during fruit–pathogen interactions? The answers to these questions will be beneficial for understanding the sophisticated regulation of ROS and effectively controlling pathogen-induced fruit decay.

## References

[1] Tian S.P., Torres R., Ballester A.R., Li B.Q., Vilanova L., González-Candelas L. (2016). Molecular aspects in pathogen-fruit interactions: Virulence and resistance. Postharvest Biol. Technol..

[2] Buron-Moles G., Torres R., Teixidó N., Usall J., Vilanova L., Viñas I. (2015). Characterisation of H_2_O_2_ production to study compatible and non-host pathogen interactions in orange and apple fruit at different maturity stages. Postharvest Biol. Technol..

[3] Heller J., Tudzynski P. (2011). Reactive oxygen species in phytopathogenic fungi: Signaling, development, and disease. Annu. Rev. Phytopathol..

[4] Camejo D., Guzmán-Cedeño A., Moreno A. (2016). Reactive oxygen species, essential molecules, during plant-pathogen interactions. Plant Physiol. Bioch..

[5] Overmyer K., Brosché M., Kangasjärvi J. (2003). Reactive oxygen species and hormonal control of cell death. Trends Plant Sci..

[6] Balaban R., Nemoto S., Finkel T. (2005). Mitochondria, oxidants, and aging. Cell.

[7] Aken O.V., Breusegem F.V. (2015). Licensed to kill: Mitochondria, chloroplasts, and cell death. Trends Plant Sci..

[8] Tian S.P., Qin G.Z., Li B.Q. (2013). Reactive oxygen species involved in regulating fruit senescence and fungal pathogenicity. Plant Mol. Biol..

[9] Kayano Y., Tanaka A., Akano F., Scott B., Takemoto D. (2013). Differential roles of NADPH oxidases and associated regulators in polarized growth, conidiation and hyphal fusion in the symbiotic fungus *Epichloë festucae*. Fungal Genet. Biol..

[10] An B., Li B.Q., Qin G.Z., Tian S.P. (2015). Function of small GTPase Rho3 in regulating growth, conidiation and virulence of *Botrytis cinerea*. Fungal Genet. Biol..

[11] Li H., Zhang Z.Q., He C., Qin G.Z., Tian S.P. (2016). Comparative proteomics reveals the potential targets of BcNoxR, a putative regulatory subunit of NADPH oxidase of *Botrytis cinerea*. Mol. Plant Microbe Interact..

[12] Pilati S., Brazzale D., Guella G., Milli A., Ruberti C., Biasioli F. (2014). The onset of grapevine berry ripening is characterized by ROS accumulation and lipoxygenase-mediated membrane peroxidation in the skin. BMC Plant Biol..

[13] Aguirre J., Ríos-Momberg M., Hewitt D., Hansberg W. (2005). Reactive oxygen species and development in microbial eukaryotes. Trends Microbiol..

[14] Fridovich I. (1995). Superoxide radical and superoxide dismutases. Annu. Rev. Biochem..

[15] Lara-Ortiz T., Riveros-Rosas H., Aguirre J. (2003). Reactive oxygen species generated by microbial NADPH oxidase NoxA regulate sexual development in *Aspergillus nidulans*. Mol. Microbiol..

[16] Giesbert S., Schürg T., Scheele S., Tudzunski P. (2008). The NADPH oxidase Cpnox1 is required for full pathogenicity of the ergot fungus *Claviceps purpurea*. Mol. Plant Pathol..

[17] Marino D., Dunand C., Puppo A., Pauly N. (2012). A burst of plant NADPH oxidases. Trends Plant Sci..

[18] Kar R.K., Gupta D.K., Palma J.M., Corpas F.J. (2015). ROS Signaling: Relevance with Site of Production and Metabolism of ROS. Reactive Oxygen Species and Oxidative Damage in Plants under Stress.

[19] Qin G.Z., Liu J., Cao B.H., Li B.Q., Tian S.P. (2011). Hydrogen peroxide acts on sensitive mitochondrial proteins to induce death of a fungal pathogen revealed by proteomic analysis. PLoS ONE.

[20] Waszczak C., Carmody M., Kangasjärvi J. (2018). Reactive oxygen species in plant signaling. Annu. Rev. Plant Biol..

[21] Apel K., Hirt H. (2004). Reactive oxygen species: Metabolism, oxidative stress, and signal transduction. Annu. Rev. Plant Biol..

[22] Jones J.D.G., Dang J.L. (2006). The plant immune system. Nature.

[23] Lester G.E., Hodges D.M. (2008). Antioxidants associated with fruit senescence and human health: Novel orange-fleshed non-netted honey dew melon genotype comparisons following different seasonal productions and cold storage durations. Postharvest Biol. Technol..

[24] Xia Y.X., Chen T., Qin G.Z., Li B.Q., Tian S.P. (2016). Synergistic action of antioxidative systems contributes to the alleviation of senescence in kiwifruit. Postharvest Biol. Technol..

[25] Qin G.Z., Meng X.H., Wang Q., Tian S.P. (2009). Oxidative damage of mitochondrial proteins contributes to fruit senescence: A redox proteomics analysis. J. Proteome Res..

[26] Qin G.Z., Wang Q., Liu J., Li B.Q., Tian S.P. (2009). Proteomic analysis of changes in mitochondrial protein expression during fruit senescence. Proteomics.

[27] Ballester A.R., Lafuente M.T., González-Candelas L. (2006). Spatial study of antioxidant enzymes, peroxidase and phenylalanine ammonia-lyase in the citrus fruit-*Penicillium digitatum* interaction. Postharvest Biol. Technol..

[28] Vilanova L., Wisniewski M., Norelli J., Vinas I., Torres R., Usall J. (2014). Transcriptomic profiling of apple in response to inoculation with a pathogen (*Penicillium expansum*) and a non-pathogen (*Penicillium digitatum*). Plant Mol. Biol. Rep..

[29] Wang Q., Lai T.F., Qin G.Z., Tian S.P. (2009). Response of Jujube fruits to exogenous oxalic acid treatment based on proteomic analysis. Plant Cell Physiol..

[30] Cai J.H., Chen J., Lu G., Zhao Y., Tian S.P., Qin G.Z. (2015). Control of brown rot on jujube and peach fruit by trisodium phosphate. Postharvest Biol. Technol..

[31] Yan F.J., Hu H., Lu L.F., Zheng X.D. (2015). Rhamnolipids induce oxidative stress responses in cherry tomato fruit to *Alternaria alternate*. Pest Manag. Sci..

[32] Ji D.C., Chen T., Ma D.Y., Liu J.L., Xu Y., Tian S.P. (2018). Inhibitory effects of methyl thujate on mycelial growth of *Botrytis cinerea* and possible mechanisms. Postharvest Biol. Technol..

[33] Liu J., Tian S.P., Meng X.H., Xu Y. (2007). Effects of chitosan on control of postharvest diseases and physiological responses of tomato fruit. Postharvest Biol. Technol..

[34] Zhang Z.Q., Chen J., Li B.Q., He C., Chen Y., Tian S.P. (2017). Influence of oxidative stress on biocontrol activity of *Cryptococcus laurentii* against blue mold on peach fruit. Front Microbiol..

[35] Yang L., Huang W., Xiong F.J., Xian Z.Q., Su D.D., Ren M.Z. (2017). Silencing of *SlPL*, which encodes a pectate lyase in tomato, confers enhanced fruit firmness, prolonged shelf-life and reduced susceptibility to grey mould. Plant Biotechnol. J..

[36] Lima-Silva V., Rosado A., Amorim-Silva V., Muñoz-Mérida A., Pons C., Bombarely A., Trelles O., Fernández-Muñoz R., Granell A., Valpuesta V. (2012). Genetic and genome-wide transcriptomic analyses identify co-regulation of oxidative response and hormone transcript abundance with vitamin C content in tomato fruit. BMC Genom..

[37] Alkan N., Fortes A.M. (2015). Insights in to molecular and metabolic events associated with fruit response to post-harvest fungal pathogens. Front. Plant Sci..

[38] Yao H.J., Tian S.P. (2005). Effects of pre-and post-harvest application of salicylic acid or methyl jasmonate on inducing disease resistance of sweet cherry fruit in storage. Postharvest Biol. Technol..

[39] Chan Z.L., Tian S.P. (2006). Induction of H_2_O_2_-metabolizing enzymes and total protein synthesis by antagonistic yeast and salicylic acid in harvested sweet cherry fruit. Postharvest Biol. Technol..

[40] Chan Z.L., Wang Q., Xu X.B., Meng X.H., Qin G.Z., Li B.Q. (2008). Functions of defense-related proteins and dehydrogenases in resistance response induced by salicylic acid in sweet cherry fruits at different maturity stages. Proteomics.

[41] Xu X.B., Tian S.P. (2008). Salicylic acid alleviated pathogen-induced oxidative stress in harvested sweet cherry fruit. Postharvest Biol. Technol..

[42] Tian S.P., Wan Y.K., Qin G.Z., Xu Y. (2006). Induction of defense responses against *Alternaria* rot by different elicitors in harvested pear fruit. Appl. Microbiol. Biotechnol..

[43] Zhu F., Chen J.J., Xiao X., Zhang M.F., Yun Z., Zeng Y.L. (2016). Salicylic acid treatment reduces the rot of postharvest citrus fruit by inducing the accumulation of H_2_O_2_, primary metabolites and lipophilic polymethoxylated flavones. Food Chem..

[44] Asghari M., Aghdam M.S. (2010). Impact of salicylic acid on post-harvest physiology of horticultural crops. Trends Food. Sci. Technol..

[45] Ge Y.H., Deng H.W., Bi Y., Li C.Y., Liu Y.Y., Dong B.Y. (2015). Postharvest ASM dipping and DPI pre-treatment regulated reactive oxygen species metabolism in muskmelon (*Cucumis melo* L.) fruit. Postharvest Biol. Technol..

[46] Gfeller A., Dubugnon L., Liechti R., Farmer E.E. (2010). Jasmonate biochemical pathway. Sci. Signal..

[47] Verma V., Ravindran P., Kumar P.P. (2016). Plant hormone-mediated regulation of stress responses. BMC Plant Biol..

[48] Zhu Z., Tian S.P. (2012). Resistant responses of tomato fruit treated with exogenous methyl jasmonate to *Botrytis cinerea* infection. Sci. Hortic. Amst..

[49] Yao H.J., Tian S.P. (2005). Effects of a biocontrol agent and methyl jasmonate on postharvest diseases of peach fruit and the possible mechanisms involved. J. Appl. Microbiol..

[50] Frenkel C., Eskin M. (1977). Ethylene evolution as related to changes in hydroperoxides in ripening tomato fruit. HortScience.

[51] Zhang Z.Q., Tian S.P., Zhu Z., Xu Y., Qin G.Z. (2012). Effects of 1-methylcyclopropene(1-MCP) on ripening and resistance of jujube (*Zizyphus jujuba* cv. Huping) fruit against postharvest disease. LWT-Food Sci. Technol..

[52] Liu R.L., Wang Y.Y., Qin G.Z., Tian S.P. (2016). Molecular basis of 1-methylcyclopropene regulating organic acid metabolism in apple fruit during storage. Postharvest Biol. Technol..

[53] Park Y.S., Im M.H., Gorinstein S. (2015). Shelf life extension and antioxidant activity of ‘Hayward’ kiwi fruit as a result of prestorage conditioning and 1-methylcyclopropene treatment. J. Food Sci. Technol..

[54] Guo M., Feng J.Z., Zhang P.Y., Jia L.Y., Chen K.S. (2014). Postharvest treatment with trans-2-hexenal induced resistance against *Botrytis cinerea* in tomato fruit. Australas. Plant Pathol..

[55] Roth U., Friebe A., Schnabl H. (2000). Resistance Induction in plants by a brassinosteroid-containing extract of *Lychnis viscaria* L.. Z. Naturforsch. C.

[56] Zhu Z., Zhang Z.Q., Qin G.Z., Tian S.P. (2010). Effects of brassinosteroids on postharvest disease and senescence of jujube fruit in storage. Postharvest Biol. Technol..

[57] Romero-Puertas M.C., Perazzolli M., Zago E.D., Delledonne M. (2004). Nitric oxide signaling functions in plant-pathogen interactions. Cell. Microbiol..

[58] Arasimowicz M., Floryszak-Wieczorek J. (2007). Nitric oxide as a bioactive signalling molecule in plant stress responses. Plant Sci..

[59] Lai T.F., Wang Y.Y., Li B.Q., Qin G.Z., Tian S.P. (2011). Defense responses of tomato fruit to exogenous nitric oxide during postharvest storage. Postharvest Biol. Technol..

[60] Vandelle E., Poinssot B., Wendehenne D., Bentéjac M., Pugin A. (2006). Integrated signaling network involving calcium, nitric oxide, and active oxygen species but not mitogen-activated protein kinases in BcPG1-elicited grapevine defenses. Mol. Plant Microbe Interact..

[61] Shi J.Y., Liu N., Gu R.X., Zhu L.Q., Zhang C., Wang Q.G. (2015). Signals induced by exogenous nitric oxide and their role in controlling brown rot disease caused by *Monilinia fructicola* in postharvest peach fruit. J. Gen. Plant Pathol..

[62] Kulik A., Noirot E., Grandperret V., Bourque S., Fromentin J., Salloignon P. (2015). Interplays between nitric oxide and reactive oxygen species in cryptogein signalling. Plant Cell Environ..

[63] Lai T.F., Chen Y., Li B.Q., Qin G.Z., Tian S.P. (2014). Mechanism of *Penicillium expansum* in response to exogenous nitric oxide based on proteomics analysis. J. Proteomics.

[64] Freschi L. (2013). Nitric oxide and phytohormone interactions: Current status and perspectives. Front. Plant Sci..

[65] Sivakumaran A., Akinyemi A., Mandon J., Cristescu S.M., Hall M.A., Harren F.J.M. (2016). ABA suppresses *Botrytis cinerea* elicited NO production in tomato to influence H_2_O_2_ generation and increase host susceptibility. Front. Plant Sci..

[66] Chung K.R. (2014). Reactive oxygen species in the citrus fungal pathogen *Alternaria alternata*: The roles of NADPH-dependent oxidase. Physiol. Mol. Plant P..

[67] Siegmund U., Marschall R., Tudzynski P. (2015). BcNoxD, a putative ER protein, is a new component of the NADPH oxidase complex in *Botrytis cinerea*. Mol. Microbiol..

[68] Segmüller N., Kokkelink L., Giesbert S., Odinius D., Kan J.V., Tudzynski P. (2008). NADPH oxidases are involved in differentiation and pathogenicity in *Botrytis cinerea*. Mol. Plant Microbe Interact..

[69] Kim H.J., Chen C.B., Kabbage M., Dickman M.B. (2011). Identification and characterization of *Sclerotinia sclerotiorum* NADPH oxidases. Appl. Environ. Microb..

[70] Semighini G.P., Harris S.D. (2008). Regulation of apical dominance in *Aspergillus nidulans* hyphae by reactive oxygen species. Genetics.

[71] Yang S.L., Chung K.R. (2012). The NADPH oxidase-mediated production of hydrogen peroxide (H_2_O_2_) and resistance to oxidative stress in the necrotrophic pathogen *Alternaria alternata* of citrus. Mol. Plant Pathol..

[72] Scott B. (2015). Conservation of fungal and animal nicotinamide adenine dinucleotide phosphate oxidase complexes. Mol. Microbiol..

[73] Galhano R., Illana A., Ryder L.S., Rodríguez-Romero J., Demuez M., Badaruddin M., Martinez-Rocha A.L., Soanes D.M., Studholme D.J., Talbot N.J. (2017). Tpc1 is an important Zn(II)_2_Cys_6_ transcriptional regulator required for polarized growth and virulence in the rice blast fungus. PLoS Pathog..

[74] Lacaze I., Lalucque H., Siegmund U., Silar P., Brun S. (2015). Identification of NoxD/Pro41 as the homologue of the p22^phox^ NADPH oxidase subunit in fungi. Mol. Microbiol..

[75] Takemoto D., Kamakura S., Saikia S., Becker Y., Wrenn R., Tanaka A. (2011). Polarity proteins Bem1 and Cdc24 are components of the filamentous fungal NADPH oxidase complex. Proc. Natl. Acad. Sci. USA.

[76] Takemoto D., Tanaka A., Scott B. (2006). A p67^Phox^-like regulator is recruited to control hyphal branching in a fungal-grass mutualistic symbiosis. Plant Cell.

[77] Boyce K.J., Hynes M.J., Andrianopoulos A. (2003). Control of morphogenesis and actin localization by the *Penicillium marneffei RAC* homolog. J. Cell Sci..

[78] Chen C.B., Dickman M.B. (2004). Dominant active Rac and dominant negative Rac revert the dominant active Ras phenotype in *Colletotrichum trifolii* by distinct signalling pathways. Mol. Microbiol..

[79] Takemoto D., Tanaka A., Scott B. (2007). NADPH oxidases in fungi: Diverse roles of reactive oxygen species in fungal cellular differentiation. Fungal Genet. Biol..

[80] Gessler N.N., Aver’yanov A.A., Belozerskaya T.A. (2007). Reactive oxygen species in regulation of fungal development. Biochemistry.

[81] Qin G.Z., Tian S.P., Chan Z.L., Li B.Q. (2007). Crucial role of antioxidant proteins and hydrolytic enzymes in pathogenicity of *Penicillium expansum*: Analysis based on proteomic approach. Mole. Cell. Proteomics.

[82] Kim K.H., Willger S.D., Park S.W., Puttikamonkul S., Grahl N., Cho Y. (2009). TmpL, a transmembrane protein required for intracellular redox homeostasis and virulence in a plant and an animal fungal pathogen. PLoS Pathog..

[83] Segal L.M., Wilson R.A. (2018). Reactive oxygen species metabolism and plant-fungal interactions. Fungal Genet. Biol..

[84] Warris A., Ballou E.R. (2018). Oxidative responses and fungal infection biology. Semin. Cell Dev. Biol..

[85] Chen C., Zhang M.K., Hu K.D., Sun K.K., Li Y.H., Hu L.Y. (2017). Deletion of Cu/Zn superoxide dismutase gene *sodC* reduces *Aspergillus niger* virulence on chinese white pear. J. Am. Soc. Hort. Sci..

[86] Tondo M.L., Petrocelli S., Ottado J., Orellano E.G. (2010). The monofunctional catalase KatE of *Xanthomonas axonopodis* pv. *citri* is required for full virulence in citrus plants. PLoS ONE.

[87] Yu C., Wang N., Wu M., Tian F., Chen H., Yang F.H. (2016). OxyR-regulated catalase CatB promotes the virulence in rice via detoxifying hydrogen peroxide in *Xanthomonas oryzae* pv. *Oryzae*. BMC Microbiol..

[88] Zhang M.K., Tang J., Huang Z.Q., Hu K.D., Li Y.H., Han Z. (2018). Deletion of the catalase gene cpeB reduces *Aspergillus niger* virulence in apple fruits. J. Agric. Food Chem..

[89] Li H., Chen Y., Zhang Z.Q., Li B.Q., Qin G.Z., Tian S.P. (2018). Pathogenic mechanisms and control strategies of *Botrytis cinerea* causing post-harvest decay in fruits and vegetables. Food Qual. Saf..

[90] Bienert G.P., Møller A.L.B., Kristiansen K.A., Schulz A., Møller I.M., Schjoerring J.K. (2007). Specific aquaporins facilitate the diffusion of hydrogen peroxide across membranes. J. Biol. Chem..

[91] Miller E.W., Dickinson B.C., Chang C.J. (2010). Aquaporin-3 mediates hydrogen peroxide uptake to regulate downstream intracellular signaling. Proc. Natl. Acad. Sci. USA.

[92] Bienert G.P., Chaumont F. (2014). Aquaporin-facilitated transmembrane diffusion of hydrogen peroxide. BBA Biomembr..

[93] Watanabe S., Moniaga C.S., Nielsen S., Hara-Chikuma M. (2016). Aquaporin-9 facilitates membrane transport of hydrogen peroxide in mammalian cells. Biochem. Bioph. Res. Commun..

[94] Sadhukhan A., Kobayashi Y., Nakano Y., Iuchi S., Kobayashi M., Sahoo L. (2017). Genome-wide association study reveals that the aquaporin NIP1; 1 contributes to variation in hydrogen peroxide sensitivity in *Arabidopsis thaliana*. Mol. Plant.

[95] Danielson J.Å., Johanson U. (2008). Unexpected complexity of the Aquaporin gene family in the moss *Physcomitrella patens*. BMC Plant Biol..

[96] Rodrigues O., Reshetnyak G., Grondin A., Saijo Y., Leonhardt N., Maurel C. (2017). Aquaporins facilitate hydrogen peroxide entry into guard cells to mediate ABA- and pathogen-triggered stomatal closure. Proc. Natl. Acad. Sci. USA.

[97] Meyers G.L., Jung K.W., Bang S., Kim J., Kim S., Hong J. (2017). The water channel protein aquaporin 1 regulates cellular metabolism and competitive fitness in a global fungal pathogen *Cryptococcus neoformans*. Environ. Microbiol. Rep..

[98] Pettersson N., Filipsson C., Becit E., Brive L., Hohmann S. (2005). Aquaporins in yeasts and filamentous fungi. Biol. Cell.

[99] An B., Li B.Q., Li H., Zhang Z.Q., Qin G.Z., Tian S.P. (2016). Aquaporin8 regulates cellular development and reactive oxygen species production, a critical component of virulence in *Botrytis cinerea*. New Phytol..

